# Free-Roaming Dog Surveys in Quito, Ecuador: Experiences, Lessons Learned, and Future Work

**DOI:** 10.3389/fvets.2021.766348

**Published:** 2021-10-28

**Authors:** Max Cárdenas, C. Jaime Grijalva, Stella de la Torre

**Affiliations:** ^1^Colegio de Ciencias Biológicas y Ambientales, Universidad San Francisco de Quito, Quito, Ecuador; ^2^Escuela de Veterinaria, Universidad San Francisco de Quito, Quito, Ecuador; ^3^Secretaría de Salud, Municipio del Distrito Metropolitano de Quito, Quito, Ecuador

**Keywords:** capture-recapture method, distance sampling, human:dog ratio, population density, abundance index

## Abstract

The selection of a survey method of free-roaming dog populations should be based on analyses of local capacities and management priorities. Here, we compare the results of surveys of the stray dog population in Quito, Ecuador, using two different methodologies and propose an alternative method for future surveys in the city. We carried out all surveys in ~5 km-transects in a sample of eight urban and eight rural parishes (16 transects total). In 2018, we used the capture-recapture method to estimate absolute population size and 95% CI. We began transect surveys at 04 h 00 (local time) and identified individuals with photographs. The main limitations of this method were errors in identifying individuals, since photographs were not always clear, partly due to low light conditions during the surveys. This method also required more time and more complex logistics. In 2019, we used distance sampling to estimate population density and began the surveys at 08 h 00 (local time). Errors in the estimation of animal-observer distances and angles were our main concern when using this method. For future surveys, we propose to carry out direct observations of dog abundance (number of free-roaming dogs/km) during street counts, complemented with capture-recapture surveys every 5 years. This alternative method albeit simple, is sensitive enough to (1) provide local authorities with objective assessments of management interventions, (2) better understanding the dynamics of free-roaming dog populations and (3) increasing public awareness about the problem of pet abandonment through citizen participation in the surveys.

## Introduction

The abandonment of dogs is a complex problem affecting animal welfare, native wildlife and public health (1). Although the magnitude of the problem and its causes may vary among regions and countries, obtaining accurate estimates of the population size and structure of free-roaming dogs is always essential to design and implement public and private interventions, and to assess their effectiveness in population control (2). Considering the complexities of surveying free-roaming animals in urban landscapes, selecting an accurate method that takes into account the socio-environmental characteristics of the urban matrix and the dog's population dynamics is of outmost importance (3). In this paper we present our experiences and learned lessons in the process of defining an adequate method for surveying and monitoring free-roaming dog populations in the Metropolitan District of Quito, the capital city of Ecuador, to (1) provide local authorities with objective assessments of management interventions, (2) better understanding the dynamics of free-roaming dog populations and (3) promote citizen participation in the surveys as means of increasing public awareness about the abandonment problem.

The need for reliable and updated information of the population status of free-roaming dogs in Quito is evidenced by the limited number of studies that have been carried out about this topic. The first estimations of the population size of dogs in the city did not provide sufficient information about the survey methods or were short term projects carried out by undergraduate students in specific sites of the urban area [e.g., (4)]. It was not until 2013 that Grijalva et al. carried out a base line estimation of free-roaming dogs in urban and rural parishes in the Quito metropolitan district using space-based random sampling procedures and the Capture—Recapture Chapman modified Lincoln-Petersen model (5). However, replicating that study was complicated because of logistic and financial constraints partly related to the limited investment in research in Ecuador (6). We believe that an effective strategy to overcome these constraints and to increase people's awareness about the problem of dog abandonment is to implement a citizen science project, with citizens actively participating in data gathering to monitor free-roaming dog populations in Quito. In 2018, we began such a project with interested citizens and personnel of public and private organizations, replicating the Capture-Recapture method used by Grijalva and collaborators in 2013 in a subsample of their surveyed areas.

The Capture-Recapture method (CR) has been used to estimate population size in several animal taxa (7). The Chapman modified Lincoln-Petersen CR model assumes a closed population and equal capture probability among animals. It requires a first survey in which animals are captured, marked and released in the population, and a second survey in which some of the captured animals are recaptures that were previously marked. The proportion of recaptured individuals is used to estimate population size (see equation in the Methods section) (8). This method has been used for estimating free-ranging dog populations in countries like Brazil (9); its limitations were analyzed by Belo et al. (1) and include the violation of the assumption of a closed population and difficulties in identifying/marking individuals.

Since we aimed to find a method that could be easily applied by volunteers to reduce errors in data collection, and that could provide adequate and sufficient information for management decisions, in 2019 we tested other method (Distance sampling). In the Distance sampling method (DS) distances to animals detected along a transect are recorded and used to estimate detection probabilities as a function of the perpendicular distances. Estimates of density are obtained based on these variables. The model assumes that all the animals on the transect are detected and that the detectability decreases with increasing distance (7). The main limitation of this method, that has not been widely applied for roaming dog populations, is the mismeasurement of distances (1).

Here, we describe the methods we used, present the survey results of each method and propose an alternative method that may be better suited for monitoring the population of free-roaming dogs in Quito.

## Materials and Methods

### Study Area

The Metropolitan District of Quito has 65 parishes (32 urban and 33 rural). It is located in the Andes at 2,850 m above sea level and has an area of 4,183 km^2^ divided in 32 urban parishes and 33 rural parishes (10). In the last national population census in 2010, there were 2,239,191 inhabitants in the district (11). In 2020, the estimated population was close to 2,800,000 inhabitants (12).

**Figure 1:**
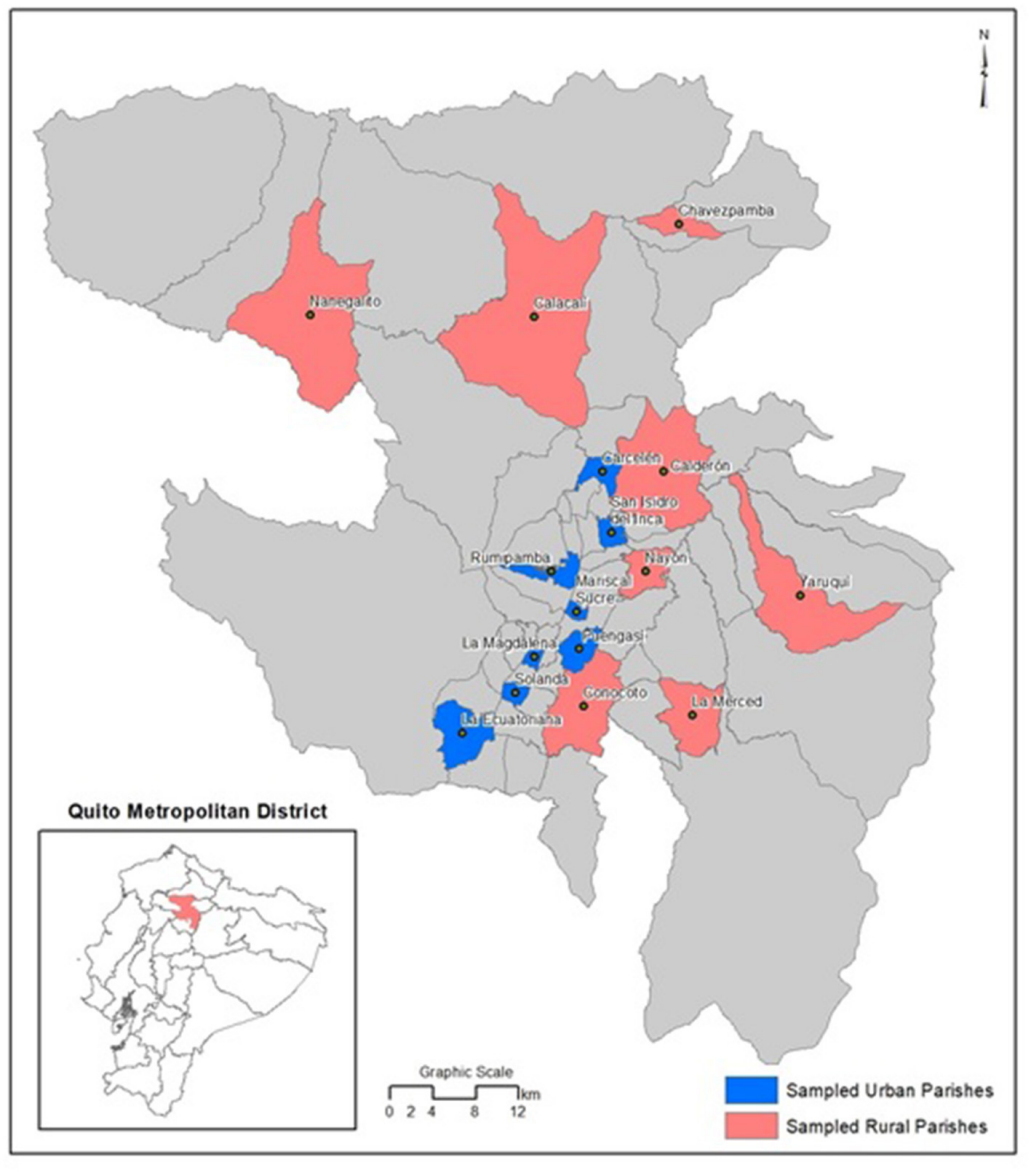
Location of the surveyed urban (in blue) and rural (in red) parishes in the Metropolitan District of Quito.

We carried out our surveys in the same 16 parishes that were surveyed by Grijalva in 2013. The parishes were selected with space-based random sampling following WSPA (13) guidelines. Eight urban parishes: Rumipamba, Mariscal Sucre, La Magdalena, La Ecuatoriana, Carcelén, San Isidro del Inca, Puengasí, Solanda, and 8 rural parishes: La Merced, Nanegalito, Chavezpamba, Yaruquí, Conocoto, Calderón, Calacalí, Nayón were selected (Figure 1). In each selected parish, 5 km transects were identified with a number in Google Earth^®^. A random number computer algorithm was used to select two sample transects per parish (5). Due to financial and logistic constraints, for the 2018 survey, in each parish we randomly selected one of the two transects used in 2013. We surveyed these same transects in the 2019 survey.

### Data Collection

In the 2018 and 2019 surveys, teams of 2 to 4 previously trained volunteers slowly walked (2 km/h) along the selected transects and used the cell phone app Survey123 for ArcGIS to record all the dogs that were on the street without a leash. All dogs were photographed, and the date, time and geographic coordinates of each sighting were automatically recorded.

In May 11th and 12th 2018, we conducted simultaneous capture-recapture surveys in each of the 16 parish ~5 km transects. We began the surveys at 04 h 00 (local time) in days with no rain. Each stray dog sighted was registered in a Survey123 form, that included a clear photograph for identification, the geographic location, and the sex of the animal. Additionally, survey teams were asked to write in a notebook a description of the color, size and notable characteristics of each animal. In the second survey, recaptured animals were identified based on similarities of photographs, written descriptions and geographic coordinates.

In November 2019, we conducted distance sampling surveys in the same ~5 km transects of the 16 parishes. We began the surveys at 08 h 00 (local time) in days with no rain. In the Survey123 form, in addition to the photograph (used to avoid double counting of individual dogs), the geographic location and the sex of the animal, we recorded a consensus estimate of the animal-observer distance (see below), the animal-observer angle (obtained with a compass or a protractor), and if it was alone or in a group. The animal-observer distance was estimated by each survey team. No measurement tools were used, but each team was trained before the survey with exercises in which they validated their distance estimates with a measuring tape. When a group of dogs was observed, a single distance was recorded for the entire group, this distance was estimated between the observer and the first observed dog. When a group of dogs was recorded, a single angle was recorded for the whole group, this angle was estimated between the observer and the first observed dog.

### Data Analysis

To estimate the free-roaming dog population in 2018, following Grijalva et al. (5), we used equation 1 (8). Where n1 is the number of animals observed on the first survey; n2 is the number of animals observed on the second survey; m2 the number of dogs observed both in the first and second surveys, and N is the total number of estimated animals.


(1) $$N=\left[\frac{\left(n1+1\right)\left(n2+1\right)}{\left(m2+1\right)}\right]-1$$

We used the two-sample method (8) to calculate the 95% confidence interval (CI) for the total count (urban and rural). We divided the estimated dog population (N) per parish by the human population of each parish recorded in the last national census in 2010 (11) to calculate the human:free-roaming dog ratio (HD ratio) per parish. We then used the median of these ratios to estimate a ratio for urban and rural parishes and a total ratio for the district (5).

In the 2019 survey, we used the animal-observer distances and the animal-observer angles to calculate the perpendicular animal-transect distances. With this new variable and the number of individuals per sighting (status: solitary/group), we ran the “Distance 7.3” software (14) to estimate the population density for the rural and urban areas. We applied uniform key; half-normal key with cosine; half-normal key with Hermite polynomial and hazard-rate key with simple polynomial models. In addition, we ran six data filters: 5% truncation, 10% truncation, 80 m truncation and 90 m truncation. The fit of each model was defined based on the Akaike Information Criteria (AIC) (14).

In addition to these analyses, we used the raw data of dog counts of the 2018 and 2019 surveys to calculate indices of abundance (number of dogs/km) in urban and rural areas by dividing the number of observed dogs in a transect by the transect length. To extend the time range of the comparison, we also calculated the abundance indices of the 2013 survey (5). For the calculation of the abundance indices in 2013 and 2018, we used the data from the first (capture) survey only.

In 2018 and 2019, we carried out a post survey workshop with the survey participants to analyze the pros and cons of the method used, recording the difficulties that the participants experienced during the surveys and the problems that the research team encountered during the data analysis.

## Results

### Capture—Recapture Method

In 2018, using the Capture—Recapture method, the estimated population of free-roaming dogs in the sample of urban parishes was 262 (95% CI 226–297), whereas in rural parishes it was 204 (95% CI 153–256). Combining urban and rural parishes, the total population was estimated in 460 (95% CI 402–517). The average HD ratio in all the surveyed parishes was 46:1 (1 dog for every 46 inhabitants). This ratio was greater in urban parishes (54:1) than in rural areas (38:1).

In the 2018 post survey workshop, the main concerns of survey participants were related to the time of the survey since low light conditions, especially from 04 h 00 through 05 h 00, made it difficult to find and photograph the dogs, affecting the reliability of the recapture events. Participants coincided in that most of the sightings occurred in the last hour of the survey, when there was more light and animals were more active.

### Distance Sampling Method

In 2019, using Distance Sampling with Hazard rate—Simple polynomial models, we estimated a mean population density of 107 dogs/km^2^ (95% CI 75–153) in the urban parishes, and of 211 dogs/km^2^ (95% CI 147–302) in the rural parishes. The mean density of free-roaming dogs, combining urban and rural parishes, was estimated in 141 dogs/ km^2^ (95% CI 109–183).

In the 2019 post survey workshop, participants commented that they were not sure about their distance estimates, especially of animals that were more than 50 m away. Some of them also mentioned they had difficulties in calculating the animal-observer angle.

### Abundance Index

The abundance indices of free-roaming dogs in urban and rural parishes were greater in 2019 (6.15 dogs/km in urban parishes and 5.41 dogs/km in rural parishes) than in 2018 and 2013 (Table 1).

**Table 1:** Index of abundance of free roaming dogs (number of dogs/km) in urban and rural parishes in Quito (indices calculated from raw data of the 2013, 2018, and 2019 surveys).

	**2013**	**2018**	**2019**
Urban parishes	2.89	4.12	8.33
Rural parishes	2.25	1.92	6.51

## Discussion

Finding an optimal method of surveying the free roaming dog population in Quito, an expanding city with an unresolved problem of pet abandonment (15, 16) is essential to provide local authorities with objective assessments of management interventions. The results of these surveys should also provide a better understanding the dynamics of stray dog populations and enhance public awareness about the problem of pet abandonment. In our citizen science study we applied the two most commonly used methods of estimating the abundance of animal populations (17) the capture-recapture method, in 2018, and line transect distance sampling, in 2019. In this section, we evaluate the feasibility of applying these methods considering the characteristics of the city environments and the conditions and resources available for research.

Given that in their baseline study of 2013, Grijalva et al. estimated the free-roaming dog population in Quito using the Capture-Recapture method (5), for our 2018 survey we decided to also use this method in a subset of their sampling areas. The comparison of the HD ratios calculated in 2013 [58:1, (5)] and 2018 (46:1), points to a 25% increase in population size in this five-years period. We are aware of the limitations of the surveys in terms of the relatively small sampling area (two and one ~5 km-transect, in 2013 and 2018, respectively, out of ~25 ~ 5 km-transects in 16 of 65 parishes); however, the fact that in both surveys there was a higher HD ratio in rural parishes than in urban parishes and a similar pattern of HD ratio differences among urban parishes, suggests that the increase in the number of free-roaming dogs in Quito is real. The reasons for this population increase may be related to a weak enforcement of the city regulations for responsible pet ownership and to limited management actions to control the stray dog population (16).

Despite the obvious convenience of using similar methods for the long term monitoring of the population, some concerns about the application of this method were raised in the post-survey discussions. The low light conditions due to the time of the surveys, affected the quality of the photos and the accuracy of the individual identification. Survey participants coincided that dogs were more active and easy to detect in the last hour of the survey, with better light conditions. In addition, because of the time of the surveys, the presence of police officers in all the survey teams was a security requirement that made the logistics more complex.

When planning the 2019 survey, based on personal observations of dog abundance, we decided to begin the surveys later in the day, at 08 h 00. We also simplified the logistics by carrying out line transect distance sampling with one survey per transect; thus devoting 1 day per transect instead of the 2 days in the capture-recapture survey. These changes facilitated dog sighting and survey organization, however, in the post-survey discussions, concerns were raised about the accuracy of the estimates of distances and angles.

To our knowledge, there is only one published study that estimated stray dog population density with distance sampling in rural villages in Philippines (18). The estimated density in that study (468 dogs/km^2^ CI 359–611) is higher than our estimated mean population density of 141 dogs/km^2^ (95% CI 109–183) in the Quito district. This could be related to differences in the socio-environmental characteristics of the study areas but we cannot exclude possible biases caused by errors in distance and angle estimations.

Evidently, given the different methods we used in the 2018 and 2019 surveys, the population estimates of both years are not comparable. However, since we used the same transects and a similar protocol to record the animals (except for the time of the surveys), we decided to calculate and compare the abundance indices of free-roaming dogs across years, including the 2013 surveys (5). Direct observations of dog abundance (number of free-roaming dogs/km) during street counts could be a good indicator of population changes to evaluate the impact of management interventions and require fewer resources than the other methods (13, 19).

The increase of the abundance indices across years agree with the trend we found when comparing the HD ratios of the 2013 and 2018 surveys, suggesting that the method, albeit simple, is sensitive enough to detect population trends. The two-fold increase from 2018 to 2019 in the abundance index of urban parishes could be partially explained by the time of the day when surveys were carried out (surveys began at 08 h 00 in 2019, whereas in 2013 and 2018 surveys began at 04 h 00), suggesting that delaying the start of the surveys to 08 h 00 enhances dog detection. The fact that the abundance index of rural parishes did not increase at the same rate, could be related to differences in the area available for the dogs to roam. This area is usually larger in rural parishes (pers. obs.) and may decrease the detectability of the animals since dogs are not restricted to the streets, as they are in most urban parishes. More surveys are needed, however, to better understand the dynamics of free-roaming dog populations in urban and rural areas.

Based on these analyses, we propose to carry out annual dog counts to calculate abundance indices in the same ~5 km transects of the 16 parishes that we have sampled in previous years. Each transect will be surveyed once by a team of 2–4 trained volunteers, walking at a pace of 2 km/h. Surveys will begin at 08 h 00 and will be carried out in the same month every year in days with no rain. Dogs sightings will be recorded in a Survey 123 form, similar to the forms we have used. We propose to maintain the citizen science approach in these future surveys since we have seen that the active participation of local people in the research allows them to better understand the problem of pet abandonment, and may facilitate their involvement in the design and implementation of actions to solve it. Data gathering with this method is less complicated, so citizen participation in the surveys will be facilitated. In addition, since implementing this method requires less resources, it may be easier for us to increase the number of sampled urban and rural parishes if we are able to obtain new funding. We propose to complement these annual direct observations of dog abundance during street counts with a capture-recapture survey every 5 years for a more complete characterization of the population dynamics. We believe this combination will allow us to better assess the effectiveness of implemented interventions and to plan future actions. We were not able to carry out the survey in 2020 because of the pandemics lock down, but we are looking forward to carry out the 2021 survey in the second semester of this year. Meanwhile, we will keep working in our education campaign to increase citizens' awareness of the pet abandonment problem, promoting the principles of responsible ownership and the adoption of rescued animals through the website https://petfriendly.usfq.edu.ec, free webinars and free neutering campaigns in rural and peri-urban areas. We are also exploring strategies to facilitate the coordination of the management interventions of public and private organizations since we strongly believe that only through long term and systematic collaborations we will eventually achieve our aim of a city with no free-roaming animals.

## Data Availability Statement

The raw data supporting the conclusions of this article will be made available by the authors, without undue reservation.

## Ethics Statement

Ethical review and approval was not required for the animal study because the study was non-invasive. Free-roaming dog surveys did not involve any type of manipulation of the animals.

## Author Contributions

All authors listed have made a substantial, direct and intellectual contribution to the work, and approved it for publication.

## Funding

This research was funded by USFQ funds for community outreach projects.

## Conflict of Interest

The authors declare that the research was conducted in the absence of any commercial or financial relationships that could be construed as a potential conflict of interest.

## Publisher's Note

All claims expressed in this article are solely those of the authors and do not necessarily represent those of their affiliated organizations, or those of the publisher, the editors and the reviewers. Any product that may be evaluated in this article, or claim that may be made by its manufacturer, is not guaranteed or endorsed by the publisher.
